# Somatostatin Receptors and Analogs in Pheochromocytoma and Paraganglioma: Old Players in a New Precision Medicine World

**DOI:** 10.3389/fendo.2021.625312

**Published:** 2021-03-29

**Authors:** Mayank Patel, Isabel Tena, Abhishek Jha, David Taieb, Karel Pacak

**Affiliations:** ^1^ Section on Medical Neuroendocrinology, Eunice Kennedy Shriver National Institute of Child Health and Human Development, National Institutes of Health, Bethesda, MD, United States; ^2^ Scientific Department, Medica Scientia Innovation Research (MedSIR), Barcelona, Spain; ^3^ Section of Medical Oncology, Consorcio Hospitalario Provincial of Castellon, Castellon, Spain; ^4^ Department of Nuclear Medicine, La Timone University Hospital, CERIMED, Aix-Marseille University, Marseille, France

**Keywords:** pheochromocytoma, paraganglioma, somatostatin receptors, somatostatin analog, peptide receptor radionuclide therapy, ^68^Ga-DOTATATE, theranostic, PET/CT

## Abstract

Neuroendocrine tumors overexpress somatostatin receptors, which serve as important and unique therapeutic targets for well-differentiated advanced disease. This overexpression is a well-established finding in gastroenteropancreatic neuroendocrine tumors which has guided new medical therapies in the administration of somatostatin analogs, both “cold”, particularly octreotide and lanreotide, and “hot” analogs, chelated to radiolabeled isotopes. The binding of these analogs to somatostatin receptors effectively suppresses excess hormone secretion and tumor cell proliferation, leading to stabilization, and in some cases, tumor shrinkage. Radioisotope-labeled somatostatin analogs are utilized for both tumor localization and peptide radionuclide therapy, with ^68^Ga-DOTATATE and ^177^Lu-DOTATATE respectively. Benign and malignant pheochromocytomas and paragangliomas also overexpress somatostatin receptors, irrespective of embryological origin. The pattern of somatostatin receptor overexpression is more prominent in *succinate dehydrogenase subunit B* gene mutation, which is more aggressive than other subgroups of this disease. While the Food and Drug Administration has approved the use of ^68^Ga-DOTATATE as a radiopharmaceutical for somatostatin receptor imaging, the use of its radiotherapeutic counterpart still needs approval beyond gastroenteropancreatic neuroendocrine tumors. Thus, patients with pheochromocytoma and paraganglioma, especially those with inoperable or metastatic diseases, depend on the clinical trials of somatostatin analogs. The review summarizes the advances in the utilization of somatostatin receptor for diagnostic and therapeutic approaches in the neuroendocrine tumor subset of pheochromocytoma and paraganglioma; we hope to provide a positive perspective in using these receptors as targets for treatment in this rare condition.

## Introduction

The theranostic revolution began over three decades ago, following the medical conception of somatostatin receptors (SSTRs) and their analogs (SSA). The identification of specific tumor targets for diagnosis and therapy of advanced diseases has been a continuing trend in oncology since its innovation. Neuroendocrine tumors (NETs) with the overexpression of SSTRs are ideal cancer models for discovering the dual ability of diagnosis and treatment using SSAs.

Two decades after the identification of somatostatin (SST) as the central regulator of neuroendocrine cell physiology in the early seventies, five SSTR subtypes were discovered (1–4). The discovery of SSTRs led to the successful introduction of somatostatin analogs (SSAs), initially as antisecretory agents, and recently as antiproliferative agents based on the results of two large phase III trials (5–7).

Through recognition of the SST molecular pathway, we can extrapolate how SSAs exert these physiologic functions. SST inhibits the secretion of neuroendocrine hormones by activating seven-transmembrane somatostatin receptors, a type of G-protein coupled receptor (GPCR). Activation of GPCR initiates a cascade of inhibiting adenyl cyclase, lowering intracellular cAMP, decreasing protein kinase A (PKA) activity, and inhibiting/activating Ca^2^ and K^+^ channels, respectively. This sequence leads to a decrease in exocytosis of peptides, effectors, or ligands resulting in a reduction of hormone secretion (8–17). SST antiproliferative effect has been much more difficult to elucidate, involving various pathways that result in a global imbalance toward increased apoptosis, cell growth modulation, and decreased angiogenesis. Besides the reduction in growth factors (GF) release, SST exerts the effect through SSTR_2_ triggering and subsequent activation of phosphotyrosine phosphatases (PTPs). This causes a downregulation of the mitogen-activated protein kinase (MAPK) pathway and of tyrosine kinase receptor (TKR) phosphorylation, inducing cell cycle arrest and decreased cell proliferation (18–27).

Moreover, clinical imaging using radiolabeled SSAs to target SSTRs, known as somatostatin receptor imaging (SRI), became a prominent method in the diagnosis and management of NETs. The earliest success of SRI was pivotal in gastroenteropancreatic (GEP)-NETs and glomus paraganglioma (PGL) localization using ^111^In-pentetreotide (Octreoscan^®^) (28, 29). The progression of SRI in NETs increased with the introduction of radiolabeled isotope ^68^Ga-SSAs for positron emission tomography (PET) imaging. Then, Lutetium-177 (^177^Lu)-SSA was developed for peptide receptor radionuclide therapy (PRRT). A particular SST-based PRRT, ^177^Lu-DOTA0-Tyr^3^-Octreotate (^177^Lu-DOTATATE), was shown to be superior to other modalities in terms of progression-free survival (PFS) in a subset of GEPNETs (30). In 2018, based on the results of the NETTER-1 trial, ^177^Lu-DOTATATE (Lutathera^®^) was approved by the FDA for foregut, midgut, and hindgut GEPNET treatment. Current management algorithms for GEPNET patients use radiolabeled, and “cold” or unlabeled SSAs for their antiproliferative and cytotoxic abilities.

**Figure 1:**
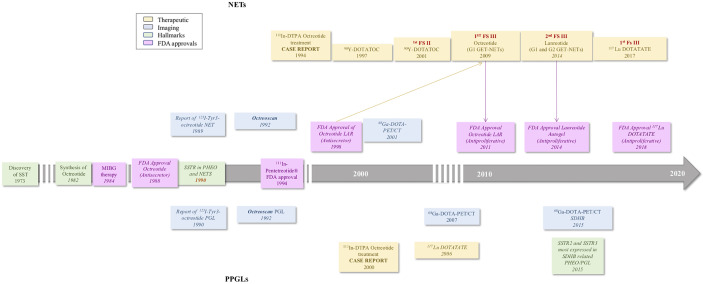
Timeline of important events in the development of somatostatin receptors and targeting analogs in neuroendocrine tumors and Pheochromocytoma and Paragangliomas (1–7, 30, 40–48).

The discovery of SSTR overexpression in pheochromocytomas and paragangliomas (PPGLs) occurred in the 1990s (31), predicting a limitless therapeutic potential of SSA; however, its role in PPGL management was not developed in parallel with GEPNETs. Initial efficacy testing of SSAs, both cold and radiolabeled, was futile, mostly due to small clinical trials without any clear accrual of therapeutic benefits (32–34) in PPGLs. Despite the therapeutic responses of SSAs in GEPNETs showing significant success (35–39), the application of cold and radiolabeled SSA in PPGL was prematurely abandoned. In the last decade, there was a rise in the use of octreotide and radiolabeled SSA for recommended therapies approved by the FDA for both functioning and nonfunctioning GEPNETs, without enough studies confirming the clinical benefits of these compounds in PPGLs for federal approval. **Figure 1** is a timeline comparing important findings and trials in SSTRs and 121 SSAs between NETs and PPGLs.

The primary therapy of choice for PPGL is surgical resection, but not in the case of unresectable advanced and metastatic tumors. A significant proportion of patients with PPGL is due to an inheritable genetic component, where the incidence of metastatic PPGL (*m*PPGL) occurs due to *succinate dehydrogenase subunit B* (*SDHB*) germline mutation patterns (49). Interestingly, *SDHB*-related PPGLs overexpress SSTRs, mainly SSTR_2_ (48). To advance and expand the clinical utilization of SSAs in this PPGL, it is imperative to view the *SDHB* subgroup as a prime example of clinical benefits that these analogs could provide.

This review summarizes the studies on the role of SSTRs focusing on PPGLs. We detail the discovery of PPGL receptors and the creation of diagnostic and therapeutic radionuclide-bound moieties to target these receptors. We also explore future perspectives for SSTRs and SSAs in driving precision-based care of PPGL patients.

## Pheochromocytoma and Paraganglioma

PPGLs are rare NETs arising from neural crest cells, specifically chromaffin cells. Differentiated based on anatomic locations, tumors from the adrenal medulla are defined as pheochromocytoma (PCC), whereas tumors from the sympathetic and parasympathetic ganglia are known as paraganglioma (PGL). While both these tumors present with similar molecular findings on pathology, they vary in manifested symptoms based on their biochemical profile (50).

More than 20 susceptibility genes (*SDHA, SDHB, SDHC, SDHD, SDHAF2, FH, VHL, EPAS1, CSDE1, MAML3, RET, NF1, MAX, HRAS, TMEM127, HIF2A, PHD1/2*) indicate predisposition to PPGLs (50). *SDHB-*related PPGLs are considered aggressive, causing more than 40% of all the metastatic cases (47). The risk of metastatic progression necessitates early diagnosis and intervention for obtaining good outcome in patients. It is important to identify symptoms and perform laboratory tests using plasma or urine metanephrines to confirm the diagnosis, followed by tumor localization through imaging.

Imaging allows personalized therapy by assisting clinicians in deciding whether surgical interventions can render the patient disease-free. PPGLs occur in a wide range of anatomical locations, from the base of the skull to the bladder, making computed tomography (CT) with intravenous contrast the initial choice of imaging modality. However, magnetic resonance imaging (MRI) with or without gadolinium is recommended if there are contraindications to CT imaging, for example contrast allergy, pregnancy, young age, and surgical clip artifacts (51).

Several predictors increase the risk of metastases: PPGL tumor > 5 cm, noradrenergic phenotype, dopaminergic phenotype, familial PPGLs (especially *SDHB* and *SDHA*), young age at initial diagnosis, multiple tumors, and recurrent disease (52, 53). PPGLs are more likely to metastasize to the lungs, liver, bones, and lymph nodes (54). While MRI has high sensitivity and specificity for PPGLs, functional imaging has shown to surpass it (55, 56). The advent of functional imaging utilizing SSTRs dramatically improved PPGL localization and identification, enabling clinicians to guide precision medicine.

## Advent of SSTR-Based Imaging in Pheochromocytoma and Paraganglioma

Success in nuclear imaging of PPGLs was achieved in 1990, when Lamberts et al. conducted a study on three NETs, including one PGL, by labeling Tyr^3^-octreotide with radioisotope ^123^Iodine (^123^I-Tyr^3^-octreotide) to target SSTRs and capturing them using gamma cameras to produce single photon emission computed tomography (SPECT) and planar images. Results showed that 29 of the 31 possible PGLs were identified, and the two missed lesions were less than 5 mm in size (40). Although it was a relatively small study in terms of patient number, these findings on SRI-related PGLs could not be ignored. Subsequent studies improved the radiolabeled nucleotide by substituting ^123^Iodide with ^111^Indium in octreotide (^111^In-pentetreotide), chelated by a diethylene triamine penta-acetic acid (DTPA) group, thus solving the problems of short half-life half-life: 13 hours for ^123^Iodide versus 24-48 hours for ^111^Indium and obscured pathology identification due to biliary excretion with subsequent accumulation in the intestines (57). A study detected 94% of PGLs in 25 patients, and an additional 36% of tumors that were not recognized with conventional imaging [CT, ultrasound, ^123^I-metaiodobenzylguanidine (^123^I-MIBG), MRI, and bone scanning] were detected using ^111^In-pentetreotide. The study showed that using ^111^In-pentetreotide could identify the PGLs identified by conventional imaging and others that were not initially visualized (58). In PCCs, ^123^I-MIBG significantly outperformed ^111^In-pentetreotide in detection (57). ^111^In-pentetreotide had higher sensitivity than ^123^I-MIBG in detecting head and neck PGLs (HNPGLs) (59–61) and mPPGL (62, 63). The ability of ^111^In-pentetreotide to bind with SSTRs, especially SSTR_2_, provided an additional diagnostic tool for clinicians to identify PPGL; however, their sole gamma-emitting capability allows the application of only SPECT to visualize them. SPECT images do not provide spatial resolution to pinpoint the precise anatomical location of PPGL.

## ^68^Ga-Based-SSA: A Preferred Imaging Radioisotope in PPGL

**Figure 2:**
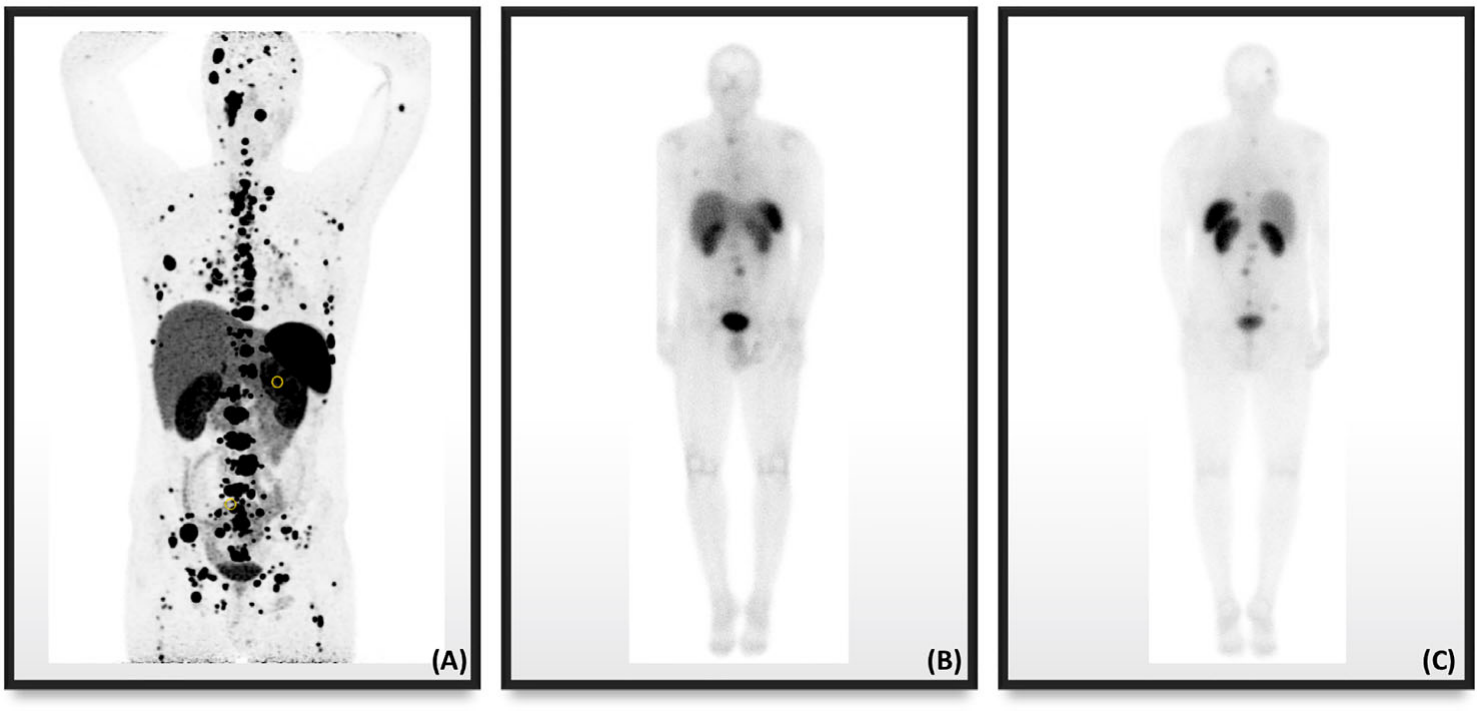
Nuclear imaging in a male patient with metastatic PPGL in the setting of *SDHD* showing the superiority in resolution of **(A)** ^68^Ga-DOTATATE PET/CT compared to Octreoscan^®^ in the **(B)** anterior anatomical plane and **(C)** posterior anatomical plane.

PET, which captures emitted positrons from radiotracers and combines them with low dose CT (PET-CT) for targeted receptor localization, was developed in the late nineties (64). Not only does PET have better spatial resolution than SPECT, it can also quantify radiotracer uptake in the form of a standardized uptake value (SUV) (65). To utilize PET-CT hybridized imaging, radiotracers -emitting positrons and targeting SSTRs were created. The first discovered radiotracer was a somatostatin analog 1-Nal3-octreotide (NOC) combined with ^68^Gallium (^68^Ga)-labeled 1,4,7,10-tetraazacyclododecane-1,4,7,10-tetraacetic acid (DOTA), better known as ^68^Ga-DOTANOC. ^68^Ga-DOTANOC targets SSTR_2,3, and 5_ (66, 67) subtypes, while another moiety, ^68^Ga-labeled DOTA-Tyr^3^-octreotide (^68^Ga-DOTATOC), showed affinity for SSTR_5_, which is not specific to PPGLs. The last moiety of the three ^68^Ga-labeled DOTA peptides is ^68^Ga-DOTA-Tyr^3^-octreotate (^68^Ga-DOTATATE), which showed a strong tendency to bind with SSTR_2_ and is ideally suited for PPGLs because of the preferential expression of these SSTR subtypes (68). These three radiolabeled somatostatin analogs were compared with previous somatostatin-targeting ^111^In-pentetreotide. The overall sensitivities for NET detection, including metastatic lesions, were much higher with ^68^Ga-labeled DOTA-peptides by PET imaging than ^111^In-pentetreotide by SPECT imaging (69–75). While these studies were not specific to PPGL tumors, one study found 16 and 12 additional PGLs on ^68^Ga-DOTATATE compared to only two on ^111^In-pentetreotide (71). The other study included two patients with PGLs, comparing ^68^Ga-DOTATOC to ^99m^Technetium-labeled hydrazinonicotinyl-Tyr^3^-octreotide (^99m^Tc-HYNIC-TOC). While the study proved that ^68^Ga-labeled DOTA-peptide was superior, individual details of these PGL patients cannot be inferred from the analysis because it was performed on a regional basis, and on other NETs (75). There were no studies comparing the efficiency of ^68^Ga-DOTATATE to that of its predecessor, ^111^In-pentetreotide, but it was widely shown to be effective in tumors that express SSTRs. In an individual case of metastatic PGL with *SDHD* germline mutation, ^68^Ga-DOTATATE PET/CT produced higher resolution of tumors than Octreoscan^®^, as seen in **Figure 2**.

Among the three radiolabeled somatostatin analogs, ^68^Ga-DOTATATE provided a brighter outlook for PPGL evaluation. SSTR expression by PPGLs, mainly extra-adrenal PGLs and *m*PPGLs, was found to be the subtype 2 variety (76). This subtype was the preferred target of ^68^Ga-DOTATATE (68). ^68^Ga-DOTATATE was shown to be superior to alternative PET radiotracers in imaging for genotypes, phenotypes, metastases, and PGL-predominant diseases. The two alternative PET radiotracers used to diagnose PPGLs, ^18^Flourine-fluorodeoxyglucose (^18^F-FDG) and ^18^F-fluorodihydroxyphenylalanine (^18^F-FDOPA), were inferior to ^68^Ga-DOTATATE in the following cohorts of patients with:

- sporadic metastatic PPGL (77)
- PGLs (78)
- HNPGLs (78, 79)
- metastatic *SDHB* PPGL (47)
- *SDHA* PPGL (80)
- *SDHD* PPGL (81)
- pediatric *SDHx* PPGL (82).

At a molecular level, the utility of ^68^Ga-based SRI in these patient cohorts can be explained by the current knowledge that *SDHx-*based lesions and extra-adrenal PGLs have higher proportions of SSTR_2_ than other PPGL types. Even though ^68^Ga-DOTATATE has lower sensitivity in other types of PPGLs than ^18^F-FDOPA, it remains the secondary radiopharmaceutical of choice in the evaluation of PPGL genotypic and phenotypic subtypes that do not fit in the cohorts mentioned above.

In two recent meta-analyses, ^68^Ga-DOTA-SSA had outperformed several radiotracers, including ^18^F-FDOPA and ^18^F-FDG. The pooled detection rate of unknown genetic mutational status in ^68^Ga-DOTA-SSA was 93% ([95% CI, 91%-95%], *P* < 0.005), higher than 80% in ^18^F-FDOPA ([95% CI, 69%–88%], *P* < 0.005) or 74% in ^18^F-FDG PET ([95% CI, 46%–91%], *P* < 0.005). The analyses showed that while genetic mutations can help select the type of radiotracers to be used in staging and diagnosing PPGL, it was not always required prior to the selection of ^68^Ga-DOTATATE, ^68^Ga-DOTATOC, and ^68^Ga-DOTANOC PET exams (83). A second meta-analysis pooled results of *m*PPGLs with germline mutational status, and the outcomes showed that ^68^Ga-DOTA-SSA PET/CT (0.97 [95% CI: 0.94-0.98]) detected more lesions than ^18^F-FDG PET/CT (0.79 [95% CI: 0.69–0.87]) (84).

**Figure 3:**
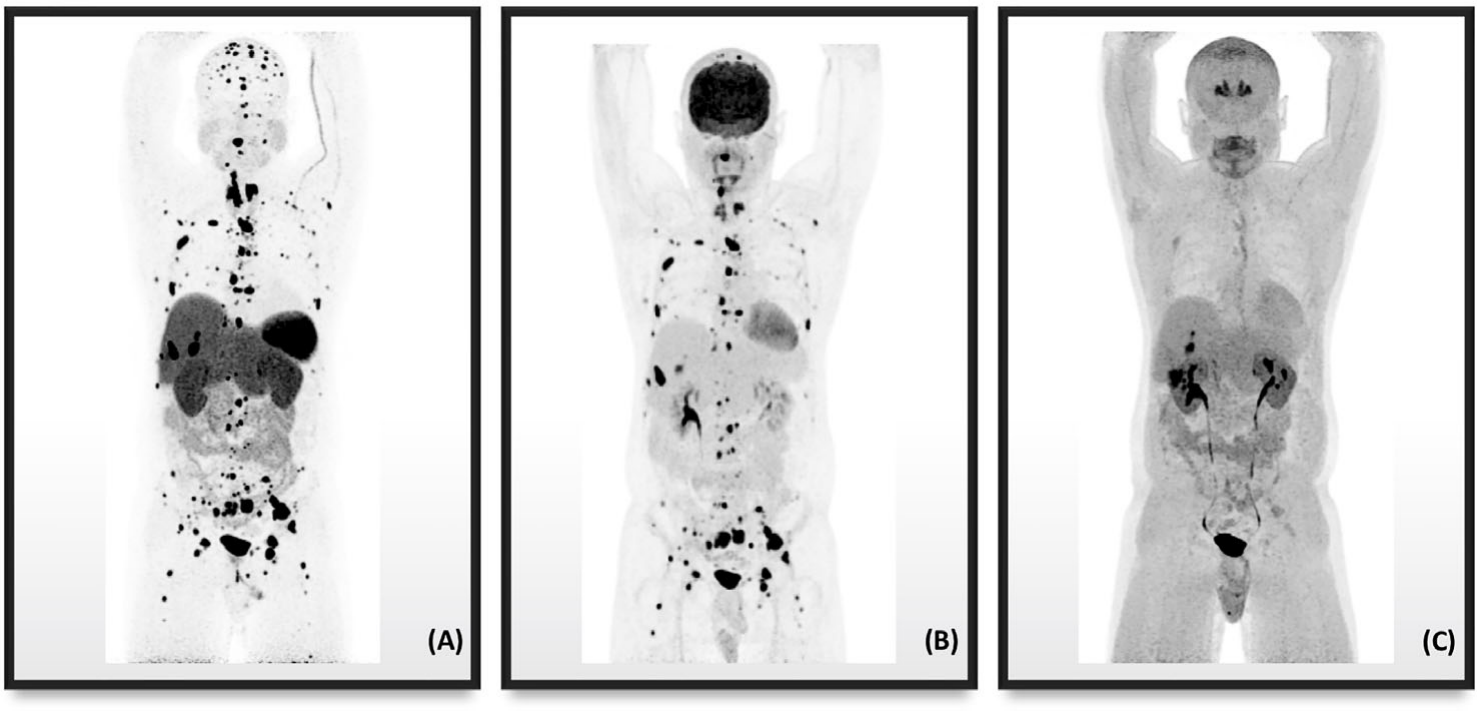
PET/CT radiotracer imaging of a 41-year-old male with metastatic PPGL in the setting of *SDHB* mutation. **(A)** ^68^Ga-DOTATATE displaying more metastatic disease than radiotracers **(B)** ^18^F-FDG and **(C)** ^18^F-FDOPA.

^68^Ga-DOTATATE PET/CT proved to be more than a complementary imaging modality to traditional CT and MRI imaging modalities. ^68^Ga-DOTATATE PET/CT has taken the place of ^111^In-pentetreotide (Octreoscan^®^) in becoming the SRI modality of choice in PPGLs, subject to the availability of a PET/CT scanner and radiotracer. It also outperformed ^18^F-FDOPA and ^18^F-FDG for detection of PGLs, *m*PGLs, HNPGLs, and *SDHx* PPGLs in adults and children. **Figure 3** illustrates the superiority of ^68^Ga-DOTATATE PET/CT compared to ^18^F-FDOPA and ^18^F-FDG of metastatic lesions in a PPGL patient with a *SDHB* mutation. While ^68^Ga-DOTATATE PET/CT effectively localizes PPGL tumors, the benefit was ultimately attributed in conversion of the ^68^Ga radiometal to a stronger beta-emitting one for therapeutic purposes.

## Experiences With Peptide Receptor Radionuclide Therapy Using Somatostatin Analogs in PPGLs

An interchange of radiolabeling on a chelated SSA (e.g., DOTA-SSA) caused a functional switch of the molecular compound from diagnostic to therapeutic capabilities. ^68^Ga-DOTA-SSA precisely located SSTRs on the surface of PPGL lesions through the capture of ^68^Ga-beta emissions by PET/CT scanners. A change in radiometal to ^177^Lutetium (^177^Lu) or ^90^Yttrium (^90^Y) gave radiolabeled DOTA-SSA the ability to emit not only imageable radiations but also deliver beta radiations to the target lesions. Lutathera^®^ was approved by the FDA for GEPNET treatment; hopefully, it is only a matter of larger-model experiences and extensive reporting until its approval in surgically unamenable or metastatic PPGLs. More trials and research are needed to determine its actual applicability in PPGLs and to support the studies mentioned in this section.

### ^177^Lu-Based-SSA PRRT

A recent 2020 report by Basu et al. reviewed 1000 patients with NETs treated with ^177^Lu-DOTATATE; 15 were diagnosed with PPGL. A particular case of metastatic HNPGL was detailed in the review, displaying stabilization of the disease after two cycles of ^177^Lu-DOTATATE on ^68^Ga-DOTATATE PET/CT (85) and should be considered in metastatic HNPGL with associated *SDHB* mutations (86). The same team recently published a retrospective study highlighting disease control of progressive *m*PGL in 6 out of 9 patients treated with ^177^Lu-DOTATATE with negative ^131^I-MIBG scans. These patients tolerated treatment without any significant adverse events (87).

A retrospective study in 2019 by Vyakaranam et al. involved 22 PPGL patients (nine with progressive disease and 13 with stable disease at the start of PRRT) and their responses to ^177^Lu-DOTATATE. The response rates of the therapy, such as biochemical response, scintigraphy, response evaluation criteria in solid tumors (RECIST), overall survival (OS), and progression-free survival (PFS) showed favorable outcomes. ^177^Lu-DOTATATE showed that only one of the 19 patients reviewed with SPECT/CT had progressive disease, while with CT, according to RECIST 1.1, all patients either had stable disease (n=20) or partial response (n=2). The median OS calculated was 49.6 months and median PFS was 21.6 (88); these were not established in other recent studies (87, 89, 90).

Another retrospective study focused on 30 patients with either parasympathetic PGL, sympathetic PGL, or PCC; after four cycles of ^177^Lu-DOTATATE, results showed either stable disease or partial response in 90% of these patients. Among these patients, 20 had progressive disease prior to the start of ^177^Lu-DOTATATE, of which 85% showed the disease controlled post-treatment (91).

### ^90^Y-Based-SSA PRRT

The alternative beta-emitting radiometal, ^90^Y, was utilized and studied in SSA-based PRRT. ^90^Y had shorter half-life, longer path length, and greater emitted energy compared to ^177^Lu (92, 93). ^90^Y also cannot be imaged using gamma cameras post-therapy because of its inherent property of being a sole beta emitter (93). With longer half-life, shorter path length, lower beta emission, and partial gamma emission, ^177^Lu had a significant advantage over ^90^Y; however, studies showed the therapeutic benefit of ^90^Y-labeled SSA as PRRT.

In a prospective study from 2019 by Kolasinska-Cwikla et al., 13 patients with metastatic *SDHB* and *SDHD* (n=5 and 8, respectively) were treated with ^90^Y-DOTATATE, with an 82% response of stable disease after 1 year. The median OS and PFS were 68 months and 35 months, respectively, with no difference in the endpoints in patients who were either secretory or non-secretory (94). A retrospective study assessing ^90^Y-DOTATATE and ^131^I-MIBG concluded that *m*PGLs were best suited for treatment by SSA-based PRRT. The study reviewed the treatment responses of 22 patients with *m*PCC or *m*PGL after three different targeted radionuclide therapies. While only two patients received ^177^Lu-DOTATATE, ^90^Y-DOTATATE performed better in terms of median PFS and RECIST 1.1 base response to treatment compared to ^131^I-MIBG (these were the two statistically significant findings) in *m*PGL with no significant difference observed when considering all the *m*PPGL patients (95).

These studies showed some positive responses to either ^177^Lu- or ^90^Y-based SSA therapy (**Table 1**, summarizing experiences using SSA-based PRRT therapies in PPGL). There are still insufficient data for FDA approval of these therapies for PPGLs.

**Table 1:** Somatostatin-based PRRT experiences with pheochromocytoma and paraganglioma in the order of year of publication (2020 to 2006).

Study Authors	Type of SSA-based PRRT	Type of study	PPGL patients	Progression at baseline	Response assessment data	Partial Responders (%)	Stable Disease(%)	Total Response (%)	PFS in months (median)	OS inmonths(median)	Concomitant Therapy
Parghane et al. (87)	^177^Lu-DOTATATE	Retrospective	9^	7	Morphological, biochemical, clinical, and SSA PET/CT	1/9 (11)	3/9 (33)	6/9 (67)	N.A.	N.A.	-
Jaiswal et al. (89)	^177^Lu-DOTATATE	Retrospective	15*	8	Morphological, biochemical, clinical, and SSA PET/CT	1/15 (7)	8/15 (53)	12/15 (80)	N.A.	N.A.	–
Roll et al. (90)^⊥^	^177^Lu-DOTATATE	Retrospective	7	1	Morphological, clinical, and SSA PET/CT	4/7 (57)	3/7 (43)	7/7 (100)	N.A.	N.A.	–
Kolasinska-Cwikla et al. (94)	^90^Y-DOTATATE	Prospective	13	13 (100%)	Morphological	1/12 (8)	9/12 (75)	10/12 (83)	35.0	68.0	–
Vyakaranam et al. (88)	^177^Lu-DOTATATE	Retrospective	22	9 (41%)	Morphological, biochemical, and clinical data	2/22 (9)	20/22 (91)	22/22 (100)	21.6	49.6	–
Zandee et al. (91)	^177^Lu-DOTATATE	Retrospective	30	20 (67%)	Morphological and clinical data	7/30 (23)	20/30 (67)	27/30 (90)	30.0	N.A.	–
Yadav et al. (96)	^177^Lu-DOTATATE	Retrospective	25	21 (84%)	SSA PET/CT, morphological, biochemical, and clinical data	7/25 (28)	14/25 (56)	21/25 (84)	32.0	N.A.	Chemotherapy (100%)
Garske-Roman et al. (97)^↲^	^177^Lu-DOTATATE	Prospective	5	2	Morphological, clinical, and biochemical data	0/5 (0)	5/5 (100)	5/5 (100)	14.0	37.0	–
Demirci et al. (98)^↲^	^177^Lu-DOTATATE	Retrospective	12	NR	Morphological and SSA PET/CT	4/8 (50)	2/8 (25)	6/8 (75)	31.4 (mean)	51.8 (mean)	–
Hamiditabar et al. (99)^↲^	^177^Lu-DOTATATE	Prospective	5	NR	Morphological, clinical, and biochemical data	0/5 (0)	4/5 (80)	4/5 (80)	N.A.	N.A.	–
Kong et al. (34)	^177^Lu-DOTATATE	Retrospective	20	6 (30%)	SSA PET/CT, morphological, biochemical, and clinical data	8/17 (47)	7/17 (42)	15/17 (88)	39.0	N.A.	Chemotherapy (45%)
Nastos et al. (95)	^177^Lu-/ ^(90)^Y-DOTATATE	Retrospective	13	13 (100%)	Morphological, biochemical, and clinical data	NR	NR	13/13 (100)	38.5	60.8	Chemotherapy, Radiation Therapy, or Cold SSA
Pinato et al. (100)	^(177)^Lu-DOTATATE	Case series	5	5 (100%)	SSA PET/CT and morphological data	1/5 (20)	3/5 (60)	4/5 (80)	17.0	N.A.	–
Estevao et al. (101)	^177^Lu-DOTATATE	Retrospective	14	4 (29%)	SSA PET/CT and clinical data	N.A.	N.A.	10/14 (71)	N.A.	N.A.	–
Puranik et al. (102)	^(177)^Lu-/^90^Y-DOTATATE/DOTATOC	Prospective	9	NR	SSA PET/CT, morphological and clinical data	4/9 (44)	5/9 (56)	9/9 (100)	N.A.	N.A.	–
Zovato et al. (33 *)*	^177^Lu-DOTATATE	Case series	4	4 (100%)	SSA scintigraphy, morphological and clinical data	2/4 (50)	2/4 (50)	4/4 (100)	N.A.	N.A.	–
Imhof et al. (103)	^90^Y-DOTATOC	Prospective	39	39 (100%)	SSA scintigraphy, morphological, biochemical, and clinical data	NR	NR	7/39 (18)	N.A.	N.A.	–
Forrer et al. (104)	^177^Lu-/^90^Y-DOTATOC	Retrospective	28	28 (100%)	Morphological, biochemical, and clinical data	7/28 (25)	13/28 (46)	20/28 (71)	N.A.	N.A.	–
van Essen et al. (45)	^177^Lu-DOTATATE	Retrospective	12	4 (33%)	Morphological, biochemical, and clinical data	2/11 (18)	6/11 (55)	8/11 (73)	N.A.	N.A.	–

^⊥^adapted from Roll et al. (90). ^↲^adapted from Satapathy et al. (105). The remaining studies were adapted from Taieb et al. (92).

^^^All 9 patients had mPGL, no PCC. *3 patients had concomitant PNETs of which 2 patients had VHL.

NR, not reported; PFS, progression-free survival; OS, overall survival.

## Clinical Side Effects of Somatostatin Analog Based Peptide Receptor Nucleotide Therapy

The clinical side effects of SSA-based PRRT include nausea, vomiting, fatigue, and abdominal pain (106). Nausea and vomiting have been attributed to commercial amino acid infusion for renal protection prior to infusion of the selected PRRT. The occurrence of nausea and vomiting can be reduced by substituting the commercial amino acid infusion with an alternative containing _L_-lysine and _L_-arginine. More serious side effects include neutropenia, lymphopenia, thrombocytopenia, and nephrotoxicity. In a review of 45 PPGL patients treated with PRRT, 3% had grade 3/4 neutropenia, 9% had thrombocytopenia, 11% had lymphopenia, and 4% had nephrotoxicity. A long-term complication of myelodysplastic syndrome was also observed in an unreported number of PPGL patients receiving the therapy (105). In a case report by Wolf et al., a dangerous side effect of Lutathera^®^ in two *m*PGL patients was hyperprogression of *m*PGL disease after three cycles of ^90^Y/^177^Lu-DOTATOC (cycle one was ^90^Y, and cycle two and three were ^177^Lu) in patient A and two cycles of ^177^Lu-DOTATATE in patient B (107). Future reporting of adverse effects of SSA-based PRRT is important in assessing the safety of this therapy in PPGL patients to determine whether the therapy can be effectuated in patients, without life-threatening side effects.

## Future Avenues of Somatostatin-Based Therapy in PPGLs

The following section will focus on ongoing studies that focus on the targeting of SSTRs by SSA based therapeutic compounds.

### Ongoing PRRT Clinical Trials

An ongoing phase II study at the National Institutes of Health (NIH), NCT03206060, could make a strong case for federal approval. The study is using Lutathera^®^ for treating progressive and inoperable PPGL patients with either germline *SDHx* mutation or sporadic disease. This prospective clinical trial will identify important clinical benefits of this treatment, focusing on the primary endpoint of PFS and several secondary endpoints, such as safety profile, OS, and quality of life. There are two other trials on Lutathera^®^ currently recruiting children (NCT03923257 in Iowa, USA) and adults (NCT04029428 in Warsaw, Poland) with nonresectable or treatment-refractory SSTR-positive PPGLs. Similar prospective clinical studies should be conducted to uncover the therapeutic potential of SSTR-targeting radiotherapy.

### Ongoing Lanreotide Clinical Trial

The long history of adoption and trial of SSA with good outcomes perpetuated an environment of ongoing clinical research and investigation. This culminated in large studies, such as the PROMID and CLARINET trials, which showed the clinical benefit of SSAs in GEPNETs (6, 7). However, the subset of NETs focused on in this review did not have extensive trials for testing the efficacy of cold SSA. There are reports of clinical stabilization of surgically unamenable PPGLs, two of which were patient experiences observed by our clinical team (80, 108), but there were no prospective or retrospective studies to either strengthen or refute these claims (80, 108–111). A prospective clinical trial (NCT03946527 in New York, USA) will evaluate the effectiveness of lanreotide in *m*PPGLs (LAMPARA) by observing tumor growth rate, overall survival, overall response rate, progression-free survival, and biochemical response.

### Next Generation Cold SSAs

Overexpression of SSTRs on the cell surfaces of PPGLs has led to ongoing investigations that target and manipulate these receptors. The antiproliferative and apoptotic effects of somatostatin and its analogs upon binding with SSTRs were identified through extensive and detailed studies (112, 113). Targeting SSTR_2_ due to their preferential expression is the current and future direction of therapeutic management in these tumors (93, 114, 115). Cold SSAs, such as octreotide and lanreotide, have a proclivity to target SSTR_2_, which have been studied and utilized in various endocrine-related diseases, including GEPNETs and acromegaly (116). Pasireotide, a second-generation SSA, targets five SSTR subtypes, unlike octreotide and lanreotide. Although it was not superior to octreotide in terms of therapy or safety profile, it could be beneficial in tumors with broader expression of SSTR subtypes, including SSTR_1_, SSTR_2_, SSTR_3_, and SSTR_5_ (117, 118). Somatoprim, another second-generation SSA, is a multi-receptor targeting analog with a preference for SSTR_2_, SSTR_4_, and SSTR_5_, which was trialed *in vitro* on growth hormone (GH)-secreting pituitary adenomas. The results showed that it had anti-secretory effects on GH adenomas that were not controlled by octreotide (119). It would be worthwhile to investigate whether somatoprim has the same antisecretory effect in PPGLs. Dopastatin, a novel chimeric analog with dual binding ability to SSTR2 and dopamine receptors (D2), also exhibited an antisecretory effect on GH in acromegaly patients (120), and antitumor effects in midgut carcinoid cells *in vitro *(121). D2 receptors were expressed in larger amounts in 52 PPGL patients than 35 GEPNET patients (122), providing another targetable receptor for dopastatin analogs through radiopeptide imaging and therapy.

### SSTR Antagonists

Development and research on SSA, which were recognized to antagonistically bind to SSTRs, are ongoing. According to an *in vitro* study by Ginj et al. (123), SSTR antagonists (SSTR-ANs) bound to NET SSTRs (especially SSTR_2_ and SSTR_3_) better than agonists but did not undergo subsequent internalization. These antagonists, sst3-ODN-8 and sst2-ANT were chelated to In by DOTA to create a receptor-targeting radioligand. These findings captured by gamma cameras were impressive in displaying antagonist-based radioligands, which bound more receptors for longer durations than their counterparts (123). The study caused a shift from the traditional theory that better binding and more benefits are derived from agonist-based analogs, mainly due to their ability to internalize the compound. A subsequent clinical comparison showed an antagonist-based SST ligand, ^111^In-DOTA-BASS, which allowed better visualization and had higher uptake in NETs than ^111^In-pentreotide (124). Based on the impressive results from first-generation SSTR-ANs, second-generation ones, such as LM3, JR10, and JR11, were developed. These second-generation SSTR-ANs were further improved in their SSTR binding capacity by using the chelator NODAGA (125). A comparative study showed that ^68^Ga-NODAGA-JR11 had higher tumoral uptake despite its lower affinity to SSTR_2_ than ^68^Ga-DOTATATE (126). The benefits were just as clear when ^177^Lu-DOTA-JR11 was used for treating four patients with 18 advanced NETs, with a ten-fold higher dose than ^177^Lu-DOTATATE and with reversible adverse events (127). A phase I/II study (NCT 02592707) focusing on the endpoints of safety, tolerability, efficacy, biodistribution, and dosimetry of ^177^Lu-OPS201 (also known as ^177^Lu-DOTA-JR11) in unresectable GEPNETs, lung carcinoids, and PPGLs is currently underway. This study could provide an additional research perspective to identify therapeutic options for PPGLs.

### Alpha Emitting ^255^Ac-DOTATATE PRRT

Alpha-emitting radiometals are also being explored in the treatment of GEPNETs and PPGLs. A study explored the utility of ^225^Actinium (^225^Ac)-DOTATATE, a targeted alpha therapy (TAT), in 32 patients with metastatic GEPNETs refractory or stable after ^177^Lu-DOTATATE therapy. Four patients with paraganglioma received TAT but were excluded from the analysis. Of the 32 GEPNET patients, 24 were assessed by RECIST 1.1 and found to have either stable or partial response. A positive biochemical response in chromogranin A (CgA) was observed as well, showing stable or decreased levels in 32 patients. There were also minimal grade III/IV toxicities reported in patients, which included gastritis in 7, weight loss in 5, flushing in 3, and headaches in 2 (128). Another study used ^225^Ac-DOTATATE as compassionate care in two patients with progressive PCC after 3 cycles of ^177^Lu-DOTATATE; however, results on the effectiveness and toxicity were not published (89).

### Cytotoxic Compounds Conjugated to SSA

Another frontier of therapeutic innovation in SSTR targeting was that of compounds linking SSA and cytotoxic agents. The SSA, Tyr^3^-octreotate, was conjugated with a microtubule-targeting agent, DM1, creating PEN-221. SSTR_2_ targeting of this agent was accomplished by the Tyr^3^-octreotate analog of the compound; after endocytosis, the DM1 portion induced a toxic payload within the targeted cells (129). A current phase I/II study (NCT 02936323) is ongoing for investigating the utility of PEN-221 in advanced NETs, including PPGLs.

## Conclusion

The “Old Players” in the title of this review shows that SSAs have a historic role in treating and managing NETs. The review hopes to restore clinical awareness of these analogs through successes achieved in PPGLs. The theranostic utility of SSAs in PPGLs can be realized once federal approval is achieved. However, research and innovation should not be halted once an approval of Lutathera^®^ for unresectable PPGLs is garnered. Research should be continued for targeting SSTRs with second-generation SSAs, SSTR-ANs, chimeric dual receptor-targeting peptides, chemotactic delivery through SSTRs, and other novel methods.

## Author Contributions

MP and IT share first co-authorship; they contributed to the conception of the idea, creation of the outline, writing, reviewing, and editing. AJ contributed to creating an outline, conceptualization, reviewing, and editing. DT contributed to reviewing. KP contributed to creating an outline, conceptualization, review, and edit of the manuscript. All authors contributed to the article and approved the submitted version.

## Funding 

This research was funded by the Eunice Kennedy Shriver National Institute of Child Health and Human Development at the National Institutes of Health.

## Conflict of Interest

The authors declare that the research was conducted in the absence of any commercial or financial relationships that could be construed as a potential conflict of interest.

The handling editor declared a past co-authorship with one of the authors KP.
